# Immunosuppressive Microenvironment Revealed by Immune Cell Landscape in Pre-metastatic Liver of Colorectal Cancer

**DOI:** 10.3389/fonc.2021.620688

**Published:** 2021-03-23

**Authors:** Dongqiang Zeng, Miaohong Wang, Jiani Wu, Siheng Lin, Zilan Ye, Rui Zhou, Gaofeng Wang, Jianhua Wu, Huiying Sun, Jianping Bin, Yulin Liao, Nailin Li, Min Shi, Wangjun Liao

**Affiliations:** ^1^Department of Oncology, Nanfang Hospital, Southern Medical University, Guangzhou, China; ^2^Department of Dermatology, Johns Hopkins School of Medicine, Baltimore, MD, United States; ^3^Department of Cardiology, State Key Laboratory of Organ Failure Research, Nanfang Hospital, Southern Medical University, Guangzhou, China; ^4^Department of Medicine-Solna, Clinical Pharmacology Group, Karolinska Institutet, Stockholm, Sweden

**Keywords:** immunosuppressive microenvironment, pre-metastatic niche, MDSC, metabolism, colorectal cancer

## Abstract

**Background:** Colorectal cancer, the fourth leading cause of cancer mortality, is prone to metastasis, especially to the liver. The pre-metastatic microenvironment comprising various resident stromal cells and immune cells is essential for metastasis. However, how the dynamic evolution of immune components facilitates pre-metastatic niche formation remains unclear.

**Methods:** Utilizing RNA-seq data from our orthotopic colorectal cancer mouse model, we applied single sample gene set enrichment analysis and Cell type Identification By Estimating Relative Subsets Of RNA Transcripts to investigate the tumor microenvironment landscape of pre-metastatic liver, and define the exact role of myeloid-derived suppressor cells (MDSCs) acting in the regulation of infiltrating immune cells and gene pathways activation. Flow cytometry analysis was conducted to quantify the MDSCs levels in human and mice samples.

**Results:** In the current work, based on the high-throughput transcriptome data, we depicted the immune cell infiltration pattern of pre-metastatic liver and highlighted MDSCs as the dominant altered cell type. Notably, flow cytometry analysis showed that high frequencies of MDSCs, was detected in the pre-metastatic liver of orthotopic colorectal cancer tumor-bearing mice, and in the peripheral blood of patients with stage I–III colorectal cancer. MDSCs accumulation in the liver drove immunosuppressive factors secretion and immune checkpoint score upregulation, consequently shaping the pre-metastatic niche with sustained immune suppression. Metabolic reprogramming such as upregulated glycolysis/gluconeogenesis and HIF-1 signaling pathways in the primary tumor was also demonstrated to correlate with MDSCs infiltration in the pre-metastatic liver. Some chemokines were identified as a potential mechanism for MDSCs recruitment.

**Conclusion:** Collectively, our study elucidates the alterations of MDSCs during pre-metastatic niche transformation, and illuminates the latent biological mechanism by which primary tumors impact MDSC aggregation in the targeted liver.

## Introduction

Distant metastasis, especially liver metastasis, is the top killer among patients with colorectal cancer (CRC). Approximately 20% of patients with CRC suffer metastatic lesions at diagnosis (1), and 50% of these ultimately develop liver metastases (2). Numerous studies have indicated that tumor metastasis is a multi-step process (3, 4); before circulating tumor cells reach the targeted organ, the preconditioned microenvironment may be reshaped to prompt cancer cell extravasation and extracellular matrix remodeling (5). This initial step, i.e., pre-metastatic niche establishment, is predominantly derived from the shift of local stromal components and recruitment of non-resident cells (6).

The dynamic differentiation and accumulation of various immune cell populations within the pre-metastatic niche collectively support immunosuppression. Although, flow cytometry remains an essential method to measure immune cell infiltration in pre-metastatic lesions despite the limited markers, the advent of a succession of emerging methodologies to address this challenge by combining high-throughput transcriptome data while determining immune cell populations. For instance, single sample gene set enrichment analysis (ssGSEA) is a gene set enrichment method that detects even minute changes in pathway activity from highly heterogeneous data (7). Cell type Identification By Estimating Relative Subsets Of RNA Transcripts (CIBERSORT) accurately quantifies immune cell type infiltration levels using a deconvolution approach (8). Given its great capacity, CIBERSORT has been applied in exploring immune infiltration of the tumor environment (9, 10).

Myeloid-derived suppressor cells (MDSCs) are a heterogeneous population differentiated from bone marrow hematopoietic stem cells, without terminally differentiating to mature granulocytes, macrophages, or dendritic cells. Under cancer and other chronic inflammation processes, MDSCs expand rapidly and can be grouped into two subsets in mice (11): CD11b^+^Ly6C^low^Ly6G^+^ polymorphonuclear myeloid suppressor cells (PMN-MDSCs) and CD11b^+^Ly6C^high^Ly6G^−^ monocyte myeloid suppressor cells (M-MDSCs). In humans, PMN-MDSCs and M-MDSCs are characterized as CD11b^+^CD14^−^CD15^+^HLA-DR^−;^ and as CD11b^+^CD14^+^CD15^−^HLA-DR^−/*low*^, respectively. Both of these include naive cell phenotypes arising from abnormal differentiation of progenitor cells under pathological conditions such as tumors or inflammation (12). Recently, ample evidence has highlighted the pivotal role of MDSCs in tumor progression via immune-suppressive mechanisms (13). Elevated MDSC levels have been detected in a variety of malignant tumors including breast cancer (14), bladder cancer (15), thyroid cancer (16), and non-small cell lung cancer (17), and are correlated with poor prognosis. During the tumor metastatic process, MDSCs migrate and aggregate in distant organs, and facilitate remodeling of the microenvironment toward a pre-metastatic niche. Prior researches on the pre-metastatic niche only focus on the role of MDSCs in hampering T cell activity (18), but how MDSCs change the components of pre-metastatic infiltrating immune cells other than CD8+ T cells is yet to be unveiled.

Taken together, utilizing RNA-seq data from orthotopic CRC mouse model, we applied ssGSEA and CIBERSORT and demonstrated the exact role of MDSCs acting in the regulation of infiltrating immune cells and gene pathways activation. Our research on the CRC pre-metastatic niche may facilitate control of cancer metastasis occurrence by targeting and harnessing MDSCs, thereby improving the prognosis of patients with colorectal cancer.

## Materials and Methods

### Patients and Blood Samples

Blood samples were collected from 25 patients with stage I-III colorectal cancer at the Nanfang Hospital (Guangzhou, China). Ten healthy volunteers were recruited to the control group. The study was approved by the Medical Ethics committee of NanFang Hospital of Southern Medical University (application approval No. NFEC-2019-263). All patients provided written informed consent before the study.

### Establishment of the Orthotopic CRC Mouse Model

All animals were obtained from the Central Laboratory of Animal Science at Southern Medical University (Guangzhou, China). All protocols for animal experiments were approved by the Nanfang Hospital Animal Ethic Committee (application approval No. NFYY-2017-128). Male BALB/c mice aged 4–6 weeks were divided into the sham-operation and tumor-bearing groups. Under anesthesia, tumor-bearing mice were disinfected and their cecum was exposed through a 5-mm incision in the left mid-abdomen. Then, 1 × 10^7^ CT26 cells were slowly inoculated into the cecal serosa. After pulling out the needle, the injected site was pressed with a sterile cotton swab for 2 min, to prevent the CT26 cell suspension from leaking into the abdominal cavity. The sham-operation group was similarly injected with 50 μL of serum-free RPMI 1640 medium. After orthotopic injection, mice were sacrificed at 3 weeks for flow-cytometric analysis and transcriptome profiling.

### Preparation of Perfusion Buffer

We prepared the perfusion buffer before the isolation experiments. To prepare perfusion buffer I, 8.0 g of NaCl, 0.122 g of NaH_2_PO_4_^*^ 2H_2_O, 0.724 g of Na_2_HPO_4_^*^ 12H_2_O, 0.4 g of KCl, 0.35 g of NaHCO_3_, 2.38 g of HEPES, 0.19 g of EGTA, 0.991 g of C_6_H_12_O_6_^*^ H_2_O, and 12,500 U of heparin sodium were weighed and dissolved in 1,000 ml of pure water; the solution was adjusted to pH 7.4 with dilute hydrochloric acid and sodium hydroxide. For perfusion buffer II, 0.029 g of CaCl_2_^*^ 2H_2_O, 1 ml of fetal bovine serum, and 0.03 g of type IV collagenase were measured and dissolved in 50 ml of high glucose DMEM medium. Finally, the above solution was filtered through a 0.22 μm filter and stored at 4°C for the subsequent experiments.

### Isolation of Immunocytes From Organs

We used an established protocol for MDSC isolation (19, 20). Peripheral blood samples from humans and mice were added with sodium citrate anticoagulant. The anticoagulant blood samples were centrifuged at 50 × g for 5 min, then the pellet was resuspended in erythrocyte lysis buffer and placed in an ice-cold box for 10 min. After centrifugation, the pellet was resuspended with PBS and could be used directly for antibody incubation. For isolating immunocytes from bone marrow, the muscle-free hind limbs of sacrificed mice were excised and the bone extremities were cut. The bone marrow was extracted in RPMI 1640 medium by washing 2–3 times with a 1 ml syringe, until the bone became white. To isolate immunocytes from the liver, kidney, and spleen, mice were anesthetized, and the liver and the inferior vena cava were exposed through a midline incision. An intravenous catheter was inserted into the inferior vena cava and fixed. The liver was sequentially perfused with perfusion buffer I and perfusion buffer II containing collagenase IV at 5 ml/min for 5 min at 37°C. After perfusion, the liver, kidney, and spleen were excised and placed on the 100-mm strainer, and smashed gently with digestive buffer. The filtered cell suspension was collected and washed 2–3 times with PBS. Immune cells were further purified by centrifugation on 30% and 70% Percoll at 1,400 × g for 20 min. The layer between 30 and 70% Percoll was aspirated carefully, and then used for the subsequent procedures.

### Flow-Cytometric Analysis of MDSCs Isolated From Organs

The cell suspensions obtained from bone marrow, liver, kidney, and spleen were centrifuged at 500 × g for 5 min. The pellet was resuspended in erythrocyte lysis buffer and placed in an ice-cold box for 10 min. After centrifugation, the pellet was resuspended with PBS. For human samples, the following antibodies were used: CD11b-APC (RanTai Co. Ltd, Shanghai, China), HLA-DR-PE (MCE, USA), CD15-FITC (Thermo Fisher Scientific, USA), CD14-PE (Thermo Fisher Scientific, USA) for 30 min on ice in the dark. For murine samples, CD11b-FITC, Ly6C-PE, Ly6G-APC antibodies (eBioscience, USA) were incubated as described above. After washing twice with 3 ml PBS, the immunocytes were analyzed on a flow cytometer (BD Biosciences, USA).

### Hematoxylin-Eosin (HE) Staining

The normal liver tissues from the sham-operation group (*n* = 5), and the matched primary tumor tissues and pre-metastatic liver tissues (*n* = 9) from the tumor-bearing group were collected and divided into 2 parts, one part was embedded in paraffin, and the other part was stored at −80°C. The dehydrated paraffin-embedded sections were dewaxed twice with xylene for 10 min and then sequentially soaked in 100, 95, 90, 80, and 70% ethyl alcohol for 5 min followed by washing with PBS for 3 min, three times. After staining with hematoxylin for 10 min and washing with running water for 5 min, 3% hydrochloric acid alcohol differentiation was performed for 2 s followed by washing back to blue for 15 min. Then, the sections were stained with eosin for 3 min, followed by soaking 70, 80, 90, 95, and 100% ethyl alcohol for 5 min and twice in xylene for 10 min. After sealing with neutral balsam, the sections were imaged using a microscope (Bx51, Olympus, Japan).

### Next-Generation RNA Sequencing

For the tissue acquisition, the mice were sacrificed at the 3 weeks after tumor cells injection. The normal liver tissues from the sham-operation group (*n* = 5), and the matched primary tumor tissues and pre-metastatic liver tissues (*n* = 9) from the tumor-bearing group were collected and divided into two parts, one part was embedded in paraffin, and the other part was stored at −80°C for RNA-sequencing analysis. The subsequent steps of RNA extraction and RNA-seq were performed by RiboBio Co. Ltd (Guangzhou, China).

### The Cancer Genome Atlas (TCGA) Gene Expression Data

Raw counts data of TCGA datasets were obtained from the UCSC Xena browser (http://xena.ucsc.edu/). RNA-seq count data were transformed into TPM using “count2tpm” function of IOBR R package (https://github.com/IOBR/IOBR) (21) to calculate the signature score and to deconvolute the immune cell fraction.

### Other Patient Cohorts Used in This Study

Seven transcriptomic data sets were enrolled in this study. GSE91061 cohort included patients with melanoma and non-small cell lung cancer treated with anti-CTLA4 and ant-PD1 therapy (22). GSE35640 cohort included patients with metastatic melanoma and non-small cell lung cancer treated with MAGE-3 agent-based immunotherapy (23). GSE115821 cohort included patients with metastatic melanoma treated with anti-PD-1 and anti-CTLA4 therapy (24). GSE63557 cohort included mouse model treated with anti-CTLA-4 therapy (25). GSE123728 cohort included patients with resectable melanoma treated with neoadjuvant PD-1 blockade therapy (26). GSE49355 cohort included patients with stage IV colorectal cancer (27). GSE14297 cohort provided transcriptomic data of primary colorectal cancers, matched liver metastases, and normal liver tissue samples (28). These transcriptomic data were downloaded from Gene Expression Omnibus (GEO) according to the accession ID.

### Functional and Pathway Enrichment Analysis

Gene annotation enrichment analysis was performed with the R package ClusterProfiler (29), Gene Ontology (GO) and Kyoto Encyclopedia of Genes and Genomes (KEGG) terms were identified with a strict cutoff of *P* < 0.01 and a false discovery rate (FDR) < 0.05.

### Immune Cell Deconvolution and Gene Signature Score Evaluation

Single sample Gene set enrichment analysis (7) was applied to evaluate the fraction of MDSCs in each tumor sample using gene sets collected from Wang et al. To quantify the proportions of immune cells in tumor and liver tissues, we used the CIBERSORT algorithm (8), and the leukocyte gene signature matrix LM22, which allows for highly sensitive and specific discrimination of 22 human immune cell phenotypes. To explore the correlation between the fraction of MDSCs and other immune escape or immune exclusion relevant biological processes, we used the gene sets (see Supplementary Table 1) curated by Mariathasan et al. (30) and Danaher et al. (31). For gene expression matrices, the expression of each gene in a signature was standardized so that its mean expression was zero, and the standard deviation across samples was 1. Then, principal component analysis (PCA) was performed, and principal component 1 was extracted to serve as the gene signature score. This approach has the advantage of focusing the score on the set with the largest block of well-correlated (or anti-correlated) genes in the set, while down-weighing the contributions from genes that do not track with other set members (30, 32).

### Statistics

The normality of variables was tested using the Shapiro-Wilk normality test (33). For comparing two groups, statistical significance for normally distributed variables was estimated using unpaired Student *t*-tests, and non-normally distributed variables were analyzed using Mann-Whitney *U* tests (also called the Wilcoxon rank-sum test). For comparisons of more than two groups, Kruskal-Wallis tests and one-way analysis of variance were used as the non-parametric and parametric methods, respectively (34). Correlation coefficients were computed by Spearman and distance correlation analyses. Cumulative survival probabilities were estimated using the Kaplan-Meier method, and were compared using the log-rank test. All heatmaps were generated using the pheatmap function (https://github.com/raivokolde/pheatmap). All statistical analyses were conducted using R (https://www.r-project.org/, version 3.50) and the *P*-values were two-sided. *P*-values < 0.05 were considered statistically significant.

## Result

### Immune Cell Landscape of Pre-metastatic Liver in the Orthotopic CRC Mouse Model

**Figure 1:**
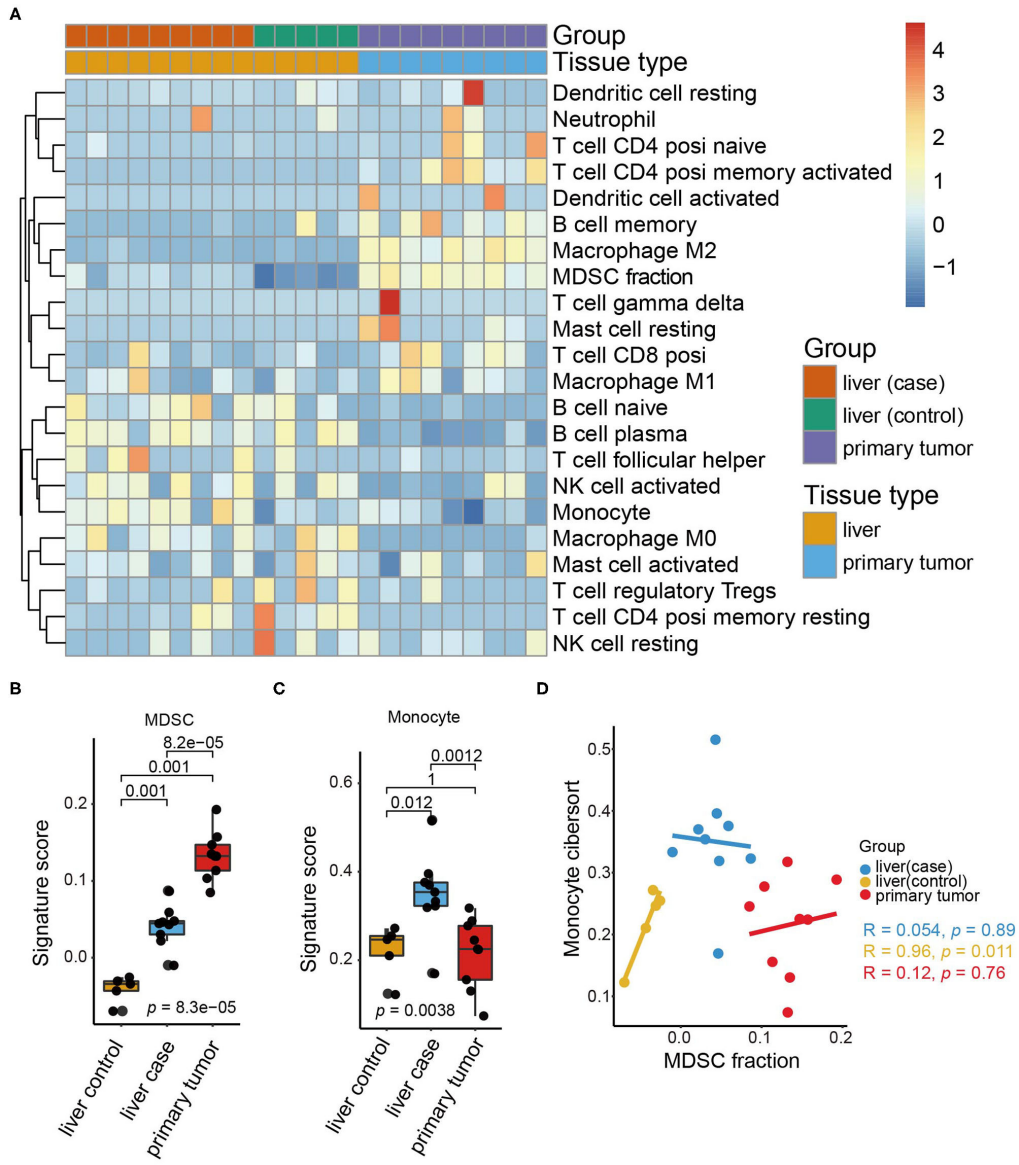
Immune cell landscape of pre-metastatic liver in the orthotopic CRC mouse model. **(A)** The expression of immune cells in our orthotopic colorectal mice models as quantified by CIBERSORT and shown by a heatmap. The tissue group and tissue type are shown as annotations. **(B)** The MDSC signature scores in the liver (control), liver (case), and primary tumor (case). The scattered dots represent the values of different samples. The thick line represents the median value, and the bottom line and top line of the boxes indicate the 25 and 75% values. The statistical difference between tissue groups was compared through the Kruskal–Wallis test. *P*-values are shown. **(C)** Monocyte signature scores in the liver (control), liver (case), and primary tumor. The statistical difference of tissue groups was compared through the Kruskal–Wallis test. *P*-values are shown. **(D)** Scatter plots depicting the correlation between the MDSC signature and monocyte signature of the liver (control), liver (case), and primary tumor, respectively. The colored dots represent the tissue groups (liver (control): yellow; liver (case): blue; primary tumor: red). Spearman correlation between the MDSC signature and monocyte signature is shown (liver (control): *p* = 0.011; liver (case): *p* = 0.89; primary tumor: *p* = 0.76).

Considering the variety of the infiltrating cell population in the pre-metastatic liver of CRC, we wondered how the underlying interactive crosstalk between different immune cells further modifies the pre-metastatic microenvironment. To better simulate the colorectal cancer microenvironment, we constructed an orthotopic CRC mouse model by orthotopically injecting CT26 cells into the intestinal mucosa of BALB/c mice. HE staining confirmed that macroscopic tumors formed after 3 weeks and without the occurrence of liver metastasis (Supplementary Figure 1). To precisely evaluate the immune cell composition of the pre-metastatic liver, we collected the primary colon tumors and matched pre-metastatic liver tissues from tumor-bearing mice, and liver tissues from sham-operation mice to conduct gene expression profiling. Analysis of the acquired RNA-seq data demonstrated the synthesized immune cell landscape of the primary tumor, pre-metastatic liver, and control liver, and revealed different immune cell infiltration patterns in the primary tumor and liver (Figure 1A).

Notably, the infiltration of M2 macrophages, MDSCs, and memory B cells increased significantly in the primary tumor, whereas the monocytes and plasma B cell counterparts were decreased. M2 macrophages and MDSCs are classical tumor-infiltrating cells releasing a variety of cytokines including growth factors, inflammatory factors, and chemokines (35), thus resulting in tumor progression and metastasis. Here, we concentrated on MDSCs and found that MDSCs accumulated predominantly in the primary tumor and less in the liver (Figure 1B).

Additionally, comparison of the immune cell composition between the pre-metastatic liver and control liver suggested that MDSCs and monocytes are the only two cell types increased in the pre-metastatic liver. Monocytes were especially enriched in the pre-metastatic liver whereas the primary tumor and control liver shared similar proportions of monocytes (Figure 1C). Despite the similar origin of monocytes and MDSCs, no statistical correlation was observed between them (Figure 1D), indicating that both cell types may influence pre-metastatic niche establishment via distinct and independent mechanisms. Higher fractions of naïve B cells, follicular helper T cells, and M2 macrophages were also observed, despite not reaching statistical significance, implying the crosstalk of MDSCs and immune cells within the pre-metastatic niche (Supplementary Figure 2A). Moreover, activated natural killer (NK) cells were negatively associated with MDSCs in the pre-metastatic niche rather than the primary tumor of the control liver tissues (*p* = 0.021, Supplementary Figure 2B) which validated the crucial role of MDSCs in reforming the pre-metastatic sites compared to other infiltrating counterparts.

### MDSCs Accumulation May Facilitate Tumor Metastasis With an Immunosuppressive Microenvironment

**Figure 2:**
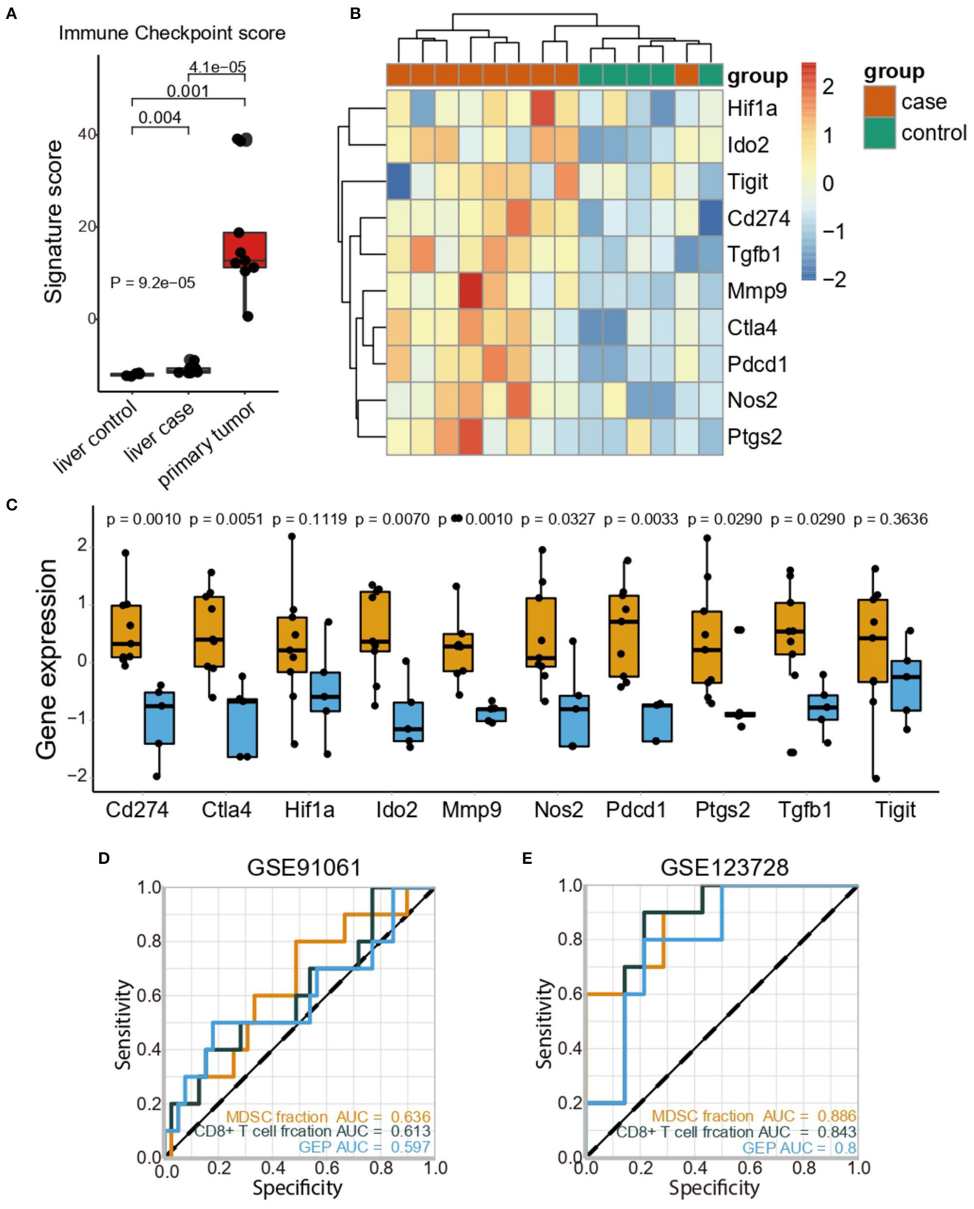
MDSCs accumulation may facilitate tumor metastasis with an immunosuppressive microenvironment. **(A)** Immune checkpoint scores in the liver (control), liver (case), and primary tumor (case). The statistical difference among tissue groups was compared by the Kruskal–Wallis test. *P*-values are shown. **(B)** Heatmap showing the expression profiles of immune checkpoint genes and immunosuppressive genes in the liver (control) and liver (case). **(C)** Expression of immunosuppressive genes in the liver (control) and liver (case). Yellow and blue represent the liver (case) and liver (control), respectively. The statistical differences between the liver (control) and liver (case) were compared by the Wilcoxon test. The box plots represent the median value and interquartile range. *P*-values are indicated. **(D)** Comparisons of the predictive accuracy of the MDSC fraction, CD8+ T cell fraction and GEP in the GSE91061 cohort. **(E)** Comparisons of the predictive accuracy of the MDSC fraction, CD8+ T cell fraction and GEP in the GSE123728 cohort.

Considering the indication that MDSCs triggered an immunosuppressive microenvironment, we further attempted to dissect the immunosuppressive mechanisms in the pre-metastatic niche from multiple facets. The high expression of negative immune checkpoint molecules is indicative of immunosuppression. We evaluated the immune checkpoint score based on immune regulatory molecules including *Cd274, Ctla4, Havcr2, Lag3, Pdcd1, Pdcd1lg2*, and *Tigit*. Consistent with the distribution of MDSC fraction, the primary tumor displayed the highest immune checkpoint scores in all groups, whereas the pre-metastatic liver showed the second highest scores (Figure 2A). Additionally, increasing levels of immune regulatory molecules like *Cd274, Pdcd1, Ctla4*, and *Tigit* were detected respectively, but no statistical significance was observed for *Tigit*. Considering the aforementioned studies, we sorted the important immunosuppressive factors secreted by MDSCs and analyzed their expression. The genes associated with T-cell suppression [*Tgfb1* (36), *Nos2* (37)] and immunosuppressive markers [*Ptgs2* (38), *Hif1a* (39)] were predominantly enriched in the pre-metastatic liver compared with the control liver (Figures 2B,C). The expression of *Mmp9* (40), which promotes aberrant vasculature formation and facilitates remodeling of the pre-metastatic niche, was higher compared to that in the control liver (Figures 2B,C).

Further investigation in The Cancer Genome Atlas Colon and Rectal Cancer (TCGA-COAD-READ) cohort confirmed that high MDSC levels were associated with the upregulation of immune checkpoint genes (Supplementary Figure 3A). Furthermore, the immune-excluded phenotype was identified as an important indicator of the immune-suppressive microenvironment; we then evaluated the corresponding signatures generated by Mariathasan et al. (30) and the exhausted CD8+ T cell signature from Danaher et al. (31). As expected, patients with CRC in the high MDSC group exhibited elevated immune-excluded signature scores, implying that MDSCs might exert immunosuppressive functions by the depletion of T cell activity and enhanced stromal cell proliferation (30) (Supplementary Figure 3B). Moreover, MDSCs were positively correlated with the marker genes for epithelial-mesenchymal transition (EMT) correlated with immune-excluded phenotypes such as TWIST1, ZEB1, ZEB2, and VIMENTIN (Supplementary Figure 3C). Considering the high correlation between MDSCs and immune signatures, we examined the predictive ability of the MDSC fraction in immune therapy cohorts (GSE91061, GSE35640, GSE115821, GSE63557, and GSE123728). Notably, the MDSC fraction discriminated the responders and non-responders toward immune checkpoint inhibitors (ICI) with a higher predictive power [area under curve (AUC) = 0.636, Figure 2D)] compared to both the CD8+ T cell fraction (AUC = 0.613) and gene expression profile score (GEP) (AUC = 0.597) in the GSE91061 cohort. The predictive value of the MDSC fraction was also validated in the GSE35640 (Supplementary Figure 4A), GSE115821 (Supplementary Figure 4B), and GSE63557 (Supplementary Figure 4C) datasets, and showed a similar predictive power compared with the CD8+ T cell fraction and GEP. Besides, high accuracy of MDSCs fraction in predicting recurrence of patients with stage III/IV melanoma undergoing neoadjuvant/adjuvant ICI therapy was observed (AUC = 0.886, Figure 2E). Collectively, MDSCs inhibited an anti-tumor immune response by upregulating the negative immune checkpoint molecules and immunosuppressive cytokines, accompanied by an immune excluded phenotype, thus reaching a convincing power in predicting the ICI efficacy.

### MDSCs Accumulated in the Pre-metastatic Liver in the Orthotopic CRC Mouse Model

**Figure 3:**
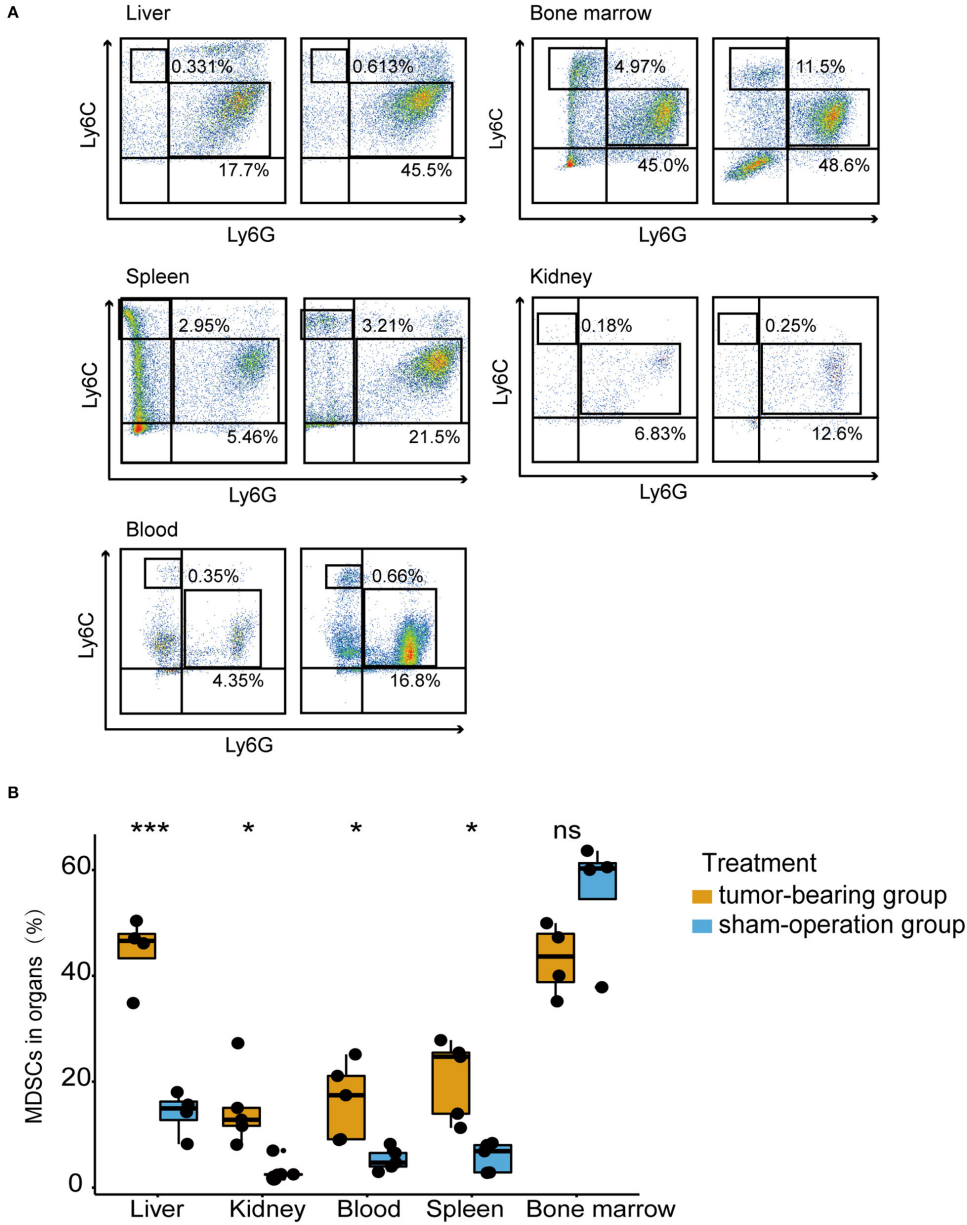
MDSCs accumulated in the pre-metastatic liver in an orthotopic CRC mouse model. **(A)** The percentage of PMN-MDSCs and M-MDSCs in the liver, bone marrow, spleen, kidney, blood of tumor-bearing mice, and sham-operation mice by flow cytometric analysis. Mouse MDSCs are classified as PMN-MDSCs (CD11b^+^Ly6C^low^Ly6G^+^) and M-MDSCs (CD11b^+^Ly6C^high^Ly6G^−^). Left figures represent the sham-operation group, and right figures represent the tumor-bearing group. *n* = 4 – 5 organ samples in each group. **(B)** Quantification of total MDSCs proportions from liver, bone marrow, spleen, kidney, blood in each treatment group. Total MDSCs are defined as the sum of PMN-MDSCs and M-MDSCs. The statistical differences between the sham-operation group and tumor-bearing group of each organ were compared by the Wilcoxon test. The box plots indicate the median value and interquartile range. ^*^P < 0.05, ^***^P < 0.001, ns indicates no significance.

Given the elevated MDSC signature score revealed in pre-metastatic liver compared to normal liver, we further verified whether high frequencies of MDSCs could be detected. Therefore, the liver, spleen, bone marrow, kidney, and peripheral blood of mouse models within 3 weeks at the pre-metastatic phase were collected for further flow cytometric analysis (Figure 3A). Total MDSCs of liver were significantly higher in the tumor-bearing group compared to sham-operation group, which was consistent with the RNA-sequence analysis results (Figure 1B). Notably, MDSCs prone to accumulate in the liver of tumor-bearing mice with the highest MDSC proportions (Figure 3B). Consistently, the fractions of MDSCs also increased in other tissues except for the bone marrow, suggesting that the liver was amenable to MDSC infiltration, thereby shaping a pre-metastatic microenvironment. As bone marrow is the major source of MDSCs, similar levels of MDSCs were observed in the normal subset and under the tumor-bearing environment (Figure 3B). Here, we also observed that higher MDSCs fractions of spleen presented in the tumor-bearing group compared to sham-operation group, indicating spleen serves as reservoir of MDSCs. As previous studies suggested (41–43), spleen was the largest immune organ which could also produce MDSCs in cancer conditions.

### High MDSC Infiltration Indicated Poor Prognosis in CRC Patients

**Figure 4:**
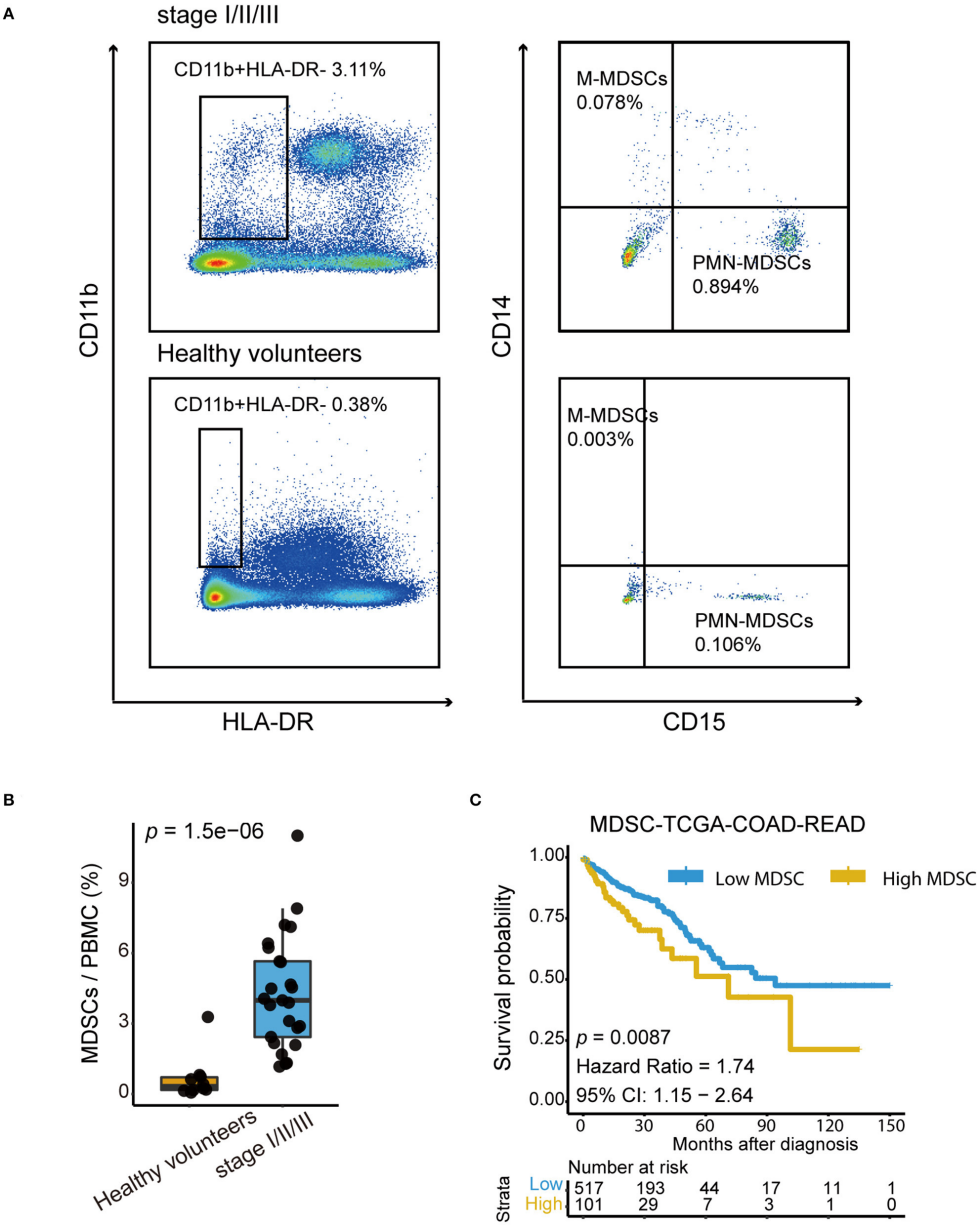
High MDSC infiltration indicated poor prognosis in CRC patients. **(A)** Representative Fluorescence activated Cell Sorting (FACs) plots showing the percentage of PMN-MDSCs and M-MDSCs in healthy volunteers and in patients with stage I–III colorectal cancer. The gating strategy of the human MDSCsubpopulation is based on CD14 and CD15 markers after the selection of CD11b and HLA-DR. **(B)** Quantification of total human MDSCs proportions in healthy volunteers and in patients with stage I-III colorectal cancer. Total MDSCs are defined as the sum of PMN-MDSCs and M-MDSCs. The statistical differences between healthy volunteers and stage I–III were compared by the Wilcoxon test. The box plots indicate the median value and interquartile range. *P*-values are shown. **(C)** Kaplan–Meier curves for high MDSC and low MDSC groups in the TCGA-COAD-READ cohort (*p* = 0.0087, Hazard Ratio = 1.74, 95% CI: 1.15 – 2.64).

To verify whether the MDSC level increases in patients with CRC but without metastasis, we first analyzed the proportion of MDSCs in the peripheral blood mononuclear cell (PBMC) fraction of healthy volunteers (*n* = 9) and patients with CRC (*n* = 25) by flow cytometry. The PBMC population was selected based on CD11b^+^ and HLA-DR^−^ (Figure 4A). We examined the proportion of PMN-MDSCs and M-MDSCs based on CD15 and CD14 expression, and defined the total MDSCs as the sum of PMN-MDSCs and M-MDSCs. Collective data revealed that the properties of total MDSCs in the PBMCs of CRC patients were elevated significantly compared with those in healthy volunteers (Figure 4B). Additionally, the association of MDSCs and prognosis was explored in the TCGA-COAD-READ cohort from TCGA using MDSC signatures proposed by Wang et.al (44). Patients with high MDSCs infiltration showed significantly worse overall survival (Figure 4C). Taken together, these results indicated that high frequencies of MDSCs in patients with CRC indicated poor clinical outcomes.

### The Primary Tumor Induced MDSC Accumulation in the Distant Liver via Metabolic Reprogramming and Chemotactic Mechanisms

**Figure 5:**
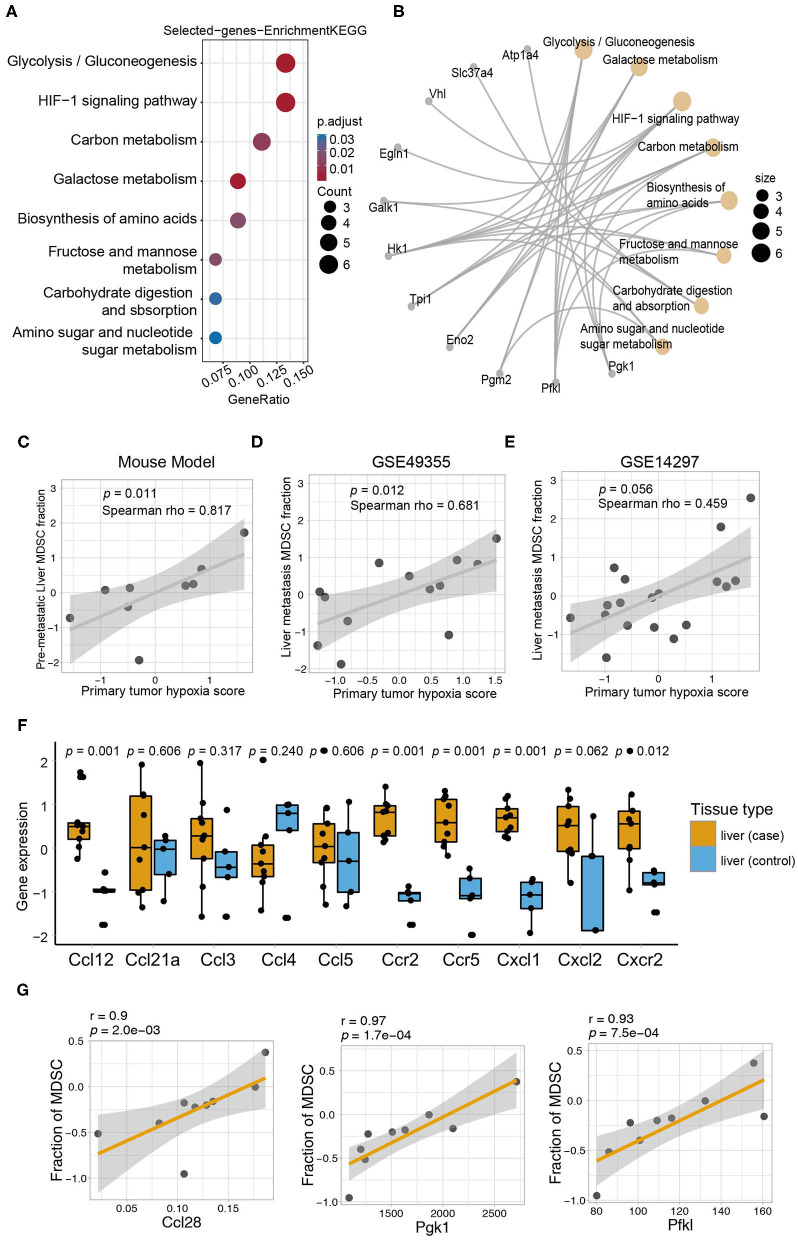
The primary tumor induced MDSC accumulation in the distant liver via metabolic reprogramming and chemotactic mechanisms. **(A)** KEGG enrichment analysis of genes correlated with MDSCs. The size of the circles represents the number of genes from each KEGG pathway. **(B)** The network showing the genes and their corresponding KEGG terms. **(C)** Correlation of the MDSC fraction from the pre-metastatic liver and hypoxia score of the primary tumor (Spearman test, *p* = 0.011, *r* = 0.817). **(D)** Correlation of the MDSC fraction in the metastatic liver and hypoxia score of the primary tumor in GSE49355 dataset (Spearman, *p* = 0.012, *r* = 0.681). **(E)** Correlation of the MDSC fraction in the metastatic liver and hypoxia score of the primary tumor in GSE14297 dataset (Spearman, *p* = 0.056, *r* = 0.459). **(F)** The expression of chemokines related to MDSCs in the liver (control) and liver (case). Yellow and blue represent the liver (case) and liver (control), respectively. Statistical differences between the liver (control) and liver (case) were compared by the Wilcoxon test. Box plots represent the median value and interquartile range. *P*-values are indicated. **(G)** The relationship between fraction of MDSC in pre-metastatic liver and chemokines (Ccl28) and HIF1 pathway genes (Pgk1 and Pfkl) in primary tumor of mouse model (Spearman, Ccl28: *r* = 0.9, *p* = 2.0*e*−03; Pgk1: *r* = 0.97, *p* = 1.7*e*−04; Pfkl: *r* = 0.93, *p* = 7.5*e*−04).

To explore the latent biological mechanism by which the primary tumor induces MDSC accumulation in pre-metastatic niches, we performed gene set enrichment analysis on genes associated with the MDSC fraction (*p* < 0.01) to perform gene set enrichment analysis (GSEA) analysis. Intriguingly, the selected genes were significantly enriched in metabolic signaling pathways including glycolysis/gluconeogenesis, HIF-1 signaling, carbon metabolism, galactose metabolism, and amino acid biosynthesis in the primary tumor, which were also associated with the MDSC infiltration level in pre-metastatic liver tissues (Figure 5A). Simultaneously, key molecules associated with the abovementioned metabolic pathways were identified. Among them, *Hk1, Pgk1, Pfkl, Eno2, Pgm2, Tpi1*, and *Galk1* were important regulating proteins involved in several metabolic pathways (Figure 5B). We further focused on the relationship between pre-metastatic MDSCs and the metabolic pathways of the primary tumor. Glucose metabolism was a vital pathway linking the primary tumor and pre-metastatic MDSC accumulation. Although glycolysis was poorly relevant to MDSCs, other pathways including the NADH metabolic process, pyruvate metabolic process, and glucose metabolic process were highly correlated with both MDSCs and glycolysis, suggesting that glucose metabolism, especially glycolysis metabolism contributed to mediate MDSC changes in the pre-metastatic microenvironment (Supplementary Figure 4D). Intriguingly, primary tumor hypoxia status was positively associated with MDSC fraction of pre-metastatic liver (mouse model: Spearman test, *r* = 0.817, *p* = 0.011; Figure 5C), while the same correlation was observed in both GSE49355 and GSE14297 datasets containing patients with colorectal cancer liver metastasis, which indicated that hypoxia condition of primary tumor might trigger the metastasis process to target liver via HIF-1 signaling (GSE49355: Spearman test, *r* = 0.681, *p* = 0.012; GSE14297: Spearman test, *r* = 0.459, *p* = 0.056, Figures 5D,E).

As chemokines are known to be essential for MDSC recruitment into the pre-metastatic microenvironment, we also analyzed the expression of chemokines that were validated to recruit MDSCs in previously published data (45). All selected chemokines except for *Ccl4* were elevated in the pre-metastatic liver, whereas only *Ccl12, Ccr2, Ccr5, Cxcl1*, and *Cxcr2* reached statistically significant levels (Figure 5F). Furthermore, we applied a correlation analysis between chemokines and MDSCs to obtain the potential chemokines acting in our model. CCL28, which mainly shows broad antimicrobial activity (46), was identified as key chemokine with a strong correlation, indicating that they may contribute in recruiting MDSCs to the pre-metastatic niche in our model (Spearman test, *p* = 0.002028, *r* = 0.9, Table 1). Except for the robust association between *Ccl28* and MDSC fraction, *Ccl28* (Figure 5G) was also observed positive correlation with immune suppressive signatures (Supplementary Figure 4E), including tumor-associated macrophages, ICI resistance, exosome assemble, and EMT process, highlighting the potential power in altering immune microenvironment of *Ccl28* is worth further investigated. In summary, metabolic signaling pathways and the CCL28 chemokine axis are prospective mechanisms that mediate the crosstalk between MDSC accumulation in the primary tumor and pre-metastatic liver.

**Table 1:** The relationship between MDSCs in pre-metastatic liver and chemokines in primary tumor of mouse model.

**Chemokines**	**Spearman correlation**	***P*-value**
Ccl21a	−0.91667	0.001312
Ccl28	0.9	0.002028
Ccr9	−0.76667	0.02139
Ccl8	−0.71667	0.036866
Cxcl14	−0.68333	0.05032
Cxcl14	−0.68333	0.05032
Ccl17	−0.6	0.096798
Ccr3	−0.58333	0.107997
Cxcl16	−0.56667	0.120574
Cxcl16	−0.56667	0.120574
Cxcl1	0.533333	0.147525
Cxcl9	−0.51667	0.1618
Ccl11	−0.5	0.177662
Ccr1	−0.48333	0.1938
Ccr5	−0.45	0.229817
Cxcl5	0.366667	0.33626
Ccr7	−0.35	0.358581
Cxcl12	−0.33333	0.385323
Ccl24	−0.3	0.436624
Ccl5	−0.3	0.436624
Ccl9	0.3	0.436624
Ccr10	0.283333	0.462991
Ccl22	−0.26667	0.493331
Ccl25	−0.26667	0.493331
Cxcl11	−0.26667	0.493331
Cxcl2	0.266667	0.493331
Ccl2	0.216667	0.580941
Ccl3	0.216667	0.580941
Ccl4	0.2	0.613404
Cxcl13	−0.2	0.613404
Ccl12	−0.16667	0.677745
Cxcl10	−0.16667	0.677745
Ccr2	−0.15	0.708069
Ccr6	−0.11667	0.775628

## Discussion

At present, it is widely accepted that prior formation of the pre-metastatic niche triggers the metastatic process. In this study, we established the orthotopic CRC mouse model and depicted the dynamic changes in immune cell composition *in vivo* by high-throughput transcriptome sequencing, then confirmed that MDSCs accumulated in the liver before metastasis. Moreover, we unveiled the metabolic pathways and chemokines involved in MDSC recruitment and in the interaction between MDSCs infiltrated in the pre-metastatic liver and primary CRC tumor. Among the various immune cells infiltrating the pre-metastatic microenvironment, our multifaceted results suggest MDSCs as the key determinant in forming the pre-metastatic microenvironment. Consistent with previous studies (47, 48), MDSCs showed great power in promoting liver metastasis, and MDSCs depletion might reverse the metastasis. However, other cell types are also indispensable. In our study, the algorithm CIBERSORT was applied based on RNA-seq data to evaluate the immune cell infiltration levels. Consequently, a whole immune cell landscape was depicted. Except for MDSCs, the amounts of monocytes were increased in the pre-metastatic liver compared to the control liver and primary tumor. Generally, monocytes are derived from hematopoietic stem cells and can differentiate into macrophages and dendritic cells (49). Emerging evidence indicates that peripheral circulating monocytes can colonize tissues where they further differentiate into macrophages to facilitate tumor metastasis (50). As our data revealed that MDSCs were not associated with monocytes, we speculated that monocytes might participate in the process of pre-metastatic niche remodeling without relying on MDSCs. An immunosuppressive microenvironment is required for attracting tumor cells. MDSCs display an exuberant potential for silencing anti-tumor immune responses by inhibiting the activities of T cells, NK cells, and dendritic cells. In our study, no significant differences in the frequencies of T cells, NK cells, and dendritic cells were observed between the pre-metastatic liver and control liver. However, the activated NK cells were negatively associated with MDSCs in the pre-metastatic niche, thus exhibiting the strong inhibitory power of MDSCs.

Immune checkpoint inhibitors represent an immense breakthrough in anti-tumor therapies. Immune checkpoint molecules are markers expressed on the surface of cancer cells and immune cells and have been implicated in cancer immune surveillance. Previous studies have shown that high levels programmed death-ligand 1 (PD-L1) are expressed on MDSCs in cancer patients (17). Moreover, PD-L1 and CTLA-4 are decreased in responding patients treated with ICI (51, 52). As immune checkpoints are not limited to PD-L1 and CTLA4, they can reflect the immunosuppressive condition of the tumor environment and can predict ICI efficacy to a certain extent. Thus, we focused comprehensively on the immune checkpoint scores based on *Cd274, Tigit, Lag3*, and other checkpoints. As expected, we noticed significantly higher immune checkpoint scores in the pre-metastatic liver, implying a strong immunosuppressive microenvironment at distant sites. In addition, high MDSC infiltration was found to be associated with upregulated negative immune checkpoint molecules and the immune-excluded phenotype in the TCGA-COAD-READ cohort. Furthermore, by applying receiver operating characteristic curve analysis, we sought to prove the predictive value of the MDSCs fraction for ICI in different cohorts of patients with melanoma (22–24, 26) and in mice models with mesothelioma (25). In line with previous studies (52), our data suggested a potential function for MDSCs as biomarkers to predict ICI efficacy.

It is recognized that the crosstalk between distant metastatic sites and the primary tumor is essential for tumor diffusion and progression as well as MDSCs aggregation. Among these, signal pathways related to immunosuppression, angiogenesis, and extracellular matrix destruction have been studied in detail, but their metabolic pathways are not well-defined. Several studies showed that the enhanced metabolic abilities of proliferating cancer cells facilitate overcoming of strict pressure during the circulating metastatic process (53). Our observations that the top 8 pathways related to metabolism as identified by GO analysis were associated with MDSCs and were highly enriched in the primary tumor and pre-metastatic liver, indicates that metabolic pathways play a critical role in linking tumor cells and metastatic lesions. Recent studies have elucidated that tumor glycolysis can boost MDSC development through the AMPK-ULK1 and autophagy pathways in triple-negative breast cancer (54). Further, the HIF-1 signaling pathway is an important part of the comprehensive MDSC functional network, and can regulate glycolysis and activate immunosuppressive effects (55–57). However, the exact role of metabolic pathways in MDSCs recruitment and accumulation in the pre-metastatic microenvironment remained unclear. We provide the first evidence that tumor cells from primary lesions may prompt MDSC migration and recruitment in distant pre-metastatic sites through metabolic pathways, especially glycolysis and HIF-1 pathways. We have also found pivotal molecules involved with metabolic pathways in both the primary tumor and pre-metastatic niche. For instance, PGK1, a major enzyme regulating glycolysis, which produces ATP in cancer cells, also promotes cancer progression (58). Therefore, our study identified the metabolic mechanisms that impact MDSC accumulation in the pre-metastatic liver in the context of primary colorectal cancer, and suggest the novel function of key molecules in several star metabolic pathways and as potential targets for exploring the underlying mechanism of MDSC migration.

It has been well-established that chemokines and chemokine receptors are important mediators to recruit and direct MDSCs to pre-metastatic organs. As previously reported, tumor-associated macrophages in primary CRC tumors recruit MDSCs into the liver and generate pre-metastatic sites through the CXCL1/CXCR2 pathway (59). Yang et al. have found that CCL2 can promote MDSC migration in murine liver tumor models (60). Our sequencing data are in agreement with previous studies supporting that CCL12 (61), CCR2, CCR5, CXCL1, and CXCR2 (45) are functional contributors to pre-metastatic lesions. CCL28 is constitutively expressed by epithelial cells in the colon; it shows strong antimicrobial ability and can recruit Tregs in autoimmune diseases (46). Here, CCL28, which has not been shown to recruit MDSCs previously, was found to be positively correlated with the MDSCs signature supported by a strong coefficient, thereby complementing the existing MDSCs-recruiting chemokine profile.

Inevitably, our study has several limitations. First, we identified a few potential mechanisms involved in MDSC recruitment, and studies that are more experimental are needed to elucidate the specific signaling pathways involved. Second, the heterogeneity of the retrieved locations like tumor margin and tumor center from mice tissues is worth noticing. Considering the different conditions of immune cell infiltration in different parts of the tumor and liver, our RNA-seq may only reflect the partial situation of pre-metastatic organs. Third, though we selected 9 samples for further analysis, incorporation of mouse model samples could provide a more comprehensive and detailed characterization of the pre-metastatic niche. Finally, the dynamic change of the pre-metastatic niche to metastasis could be observed if the tumor-bearing time were to be extended appropriately.

In conclusion, we comprehensively evaluated the immune cell landscape of the pre-metastatic site and primary CRC tumor via transcriptome analysis, and highlighted MDSCs as the prominent cell type contributing to the immunosuppressive condition and generation of the pre-metastatic niche. Our study also sheds light on the potential metabolic pathways involved in MDSC regulation by the primary tumor and their recruitment into pre-metastatic organs. We offer an explanation for the link between MDSCs and CRC metastatic process, targeting MDSCs as a promising strategy for future restricting cancer metastasis therapy development.

## Data Availability Statement

The datasets presented in this study can be found in online repositories. The names of the repository/repositories and accession number(s) can be found below: https://www.ncbi.nlm.nih.gov/geo/, GSE147044.

## Ethics Statement

The studies involving human participants were reviewed and approved by Medical Ethics committee of NanFang Hospital of Southern Medical University. The patients/participants provided their written informed consent to participate in this study. The animal study was reviewed and approved by Nanfang Hospital Animal Ethic Committee.

## Author Contributions

WL and DZ conceived and designed the study. MW, JianiW, and SL performed the experiments. JianhW and GW acquired the data. DZ, RZ, and HS analyzed and interpreted the data. JianiW, DZ, and ZY drafted the manuscript. NL, YL, JB, MS, and WL revised of the manuscript for important intellectual content critically. WL obtained funding. DZ, JianiW, and MW conducted statistical analysis. All authors reviewed the article.

## Conflict of Interest

The authors declare that the research was conducted in the absence of any commercial or financial relationships that could be construed as a potential conflict of interest.
