# Supplementary material for: Immunosuppressive Microenvironment Revealed by Immune Cell Landscape in Pre-metastatic Liver of Colorectal Cancer
Source: Front Oncol. 2021 Mar 23;11:620688. doi: 10.3389/fonc.2021.620688 (PMC8021849; doi:10.3389/fonc.2021.620688)
Supplement: Supplementary file 1 [file Data_Sheet_1.pdf]

Supplementary Material

1 Supplementary Tables

1.1 Supplementary Table 1 Gene Signatures enrolled in this study

MDSC_Wang.et.al	CD_8_T_effector	Immune_Checkpoint	Pan_F_TBRs	EMT1	EMT2	EMT3
CCR2	CD8A	CD274	ACTA2	CLDN3	AXL	SOX9
CXCR4	GZMA	PDCD1LG2	ACTG2	CLDN7	ROR2	TWIST1
CXCR2	GZMB	CTLA4	ADAM12	CLDN4	WNT5A	FOXF1
ITGAM	IFNG	PDCD1	ADAM19	CDH1	LOXL2	ZEB1
ITGAX	CXCL9	LAG3	CNN1	VIM	TWIST2	ZEB2
ANPEP	CXCL10	HAVCR2	COL4A1	TWIST1	TAGLN	GATA6
CD14	PRF1	TIGIT	CTGF	ZEB1	FAP	
FUT4	TBX21		CTPS1	ZEB2		
CD33			FAM101B			
CD34			FSTL3			
CD38			HSPB1			
ENTPD1			IGFBP3			
PTPRC			PXDC1			
CEACAM8			SEMA7A			
CD80			SH3PXD2A			
CSF1R			TAGLN			
IL4R			TGFBI			
CSF3			TNS1			
CSF2			TPM1			
CXCL8						
CCL2						
TNF						
CXCL12						
CSF1R						
S100A8						
S100A9						
STAT1						
STAT3						
STAT5A						
ARG1						
NOS2						
CD274						
TLR3						
TLR4						
TGFB1						
IL10						

FOXP3
IDO1
PDCD1

2 Supplementary Figures

2.1 Supplementary Figure 1

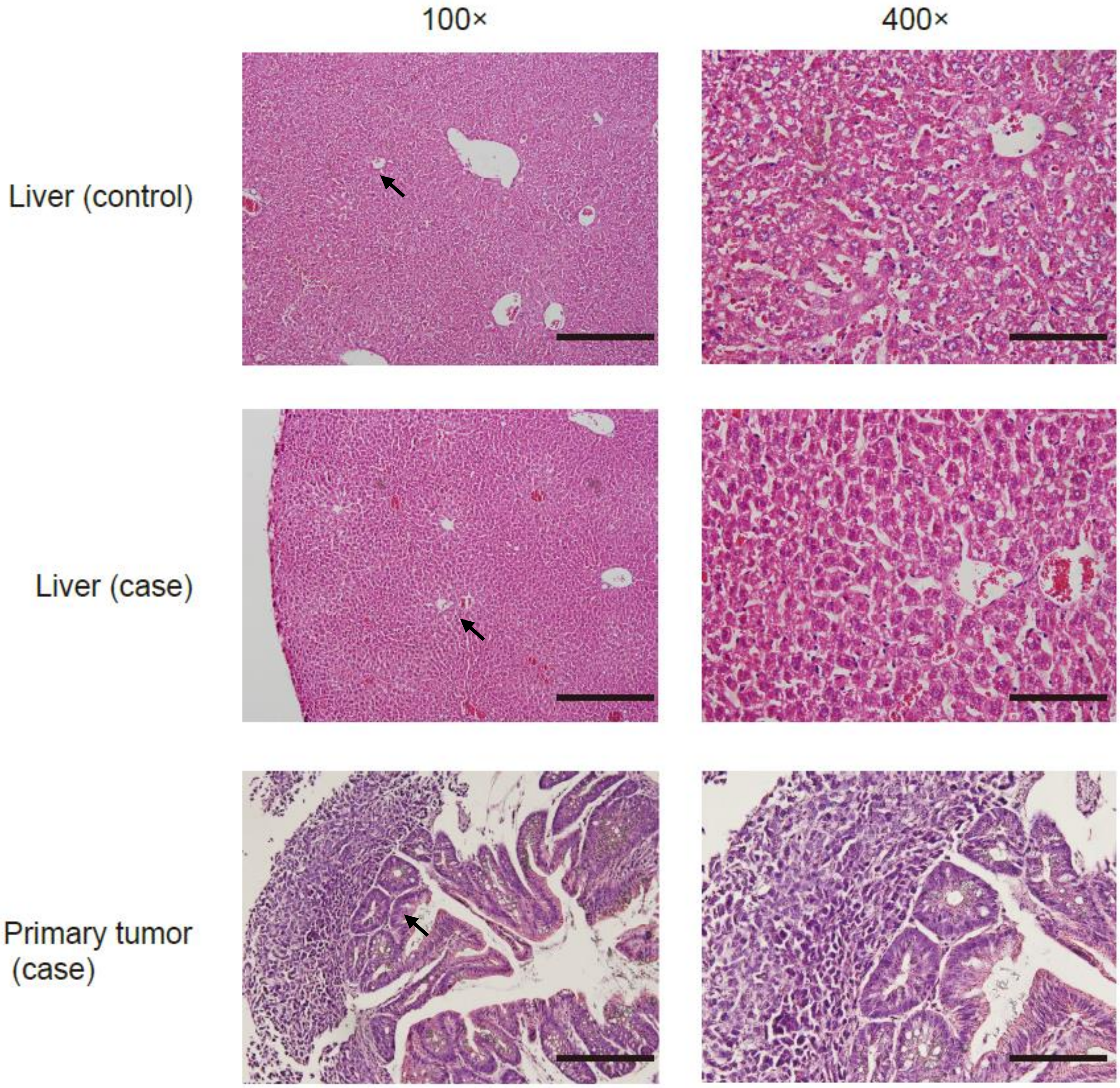

Supplementary Figure 1. HE staining of liver and tumor from orthotopic colorectal mice models. Representative photomicrographs of liver (control), liver (case), and primary tumor (case) from orthotopic colorectal mice models. Scale bar: 100 µm, 25 µm.

2.2 Supplementary Figure 2

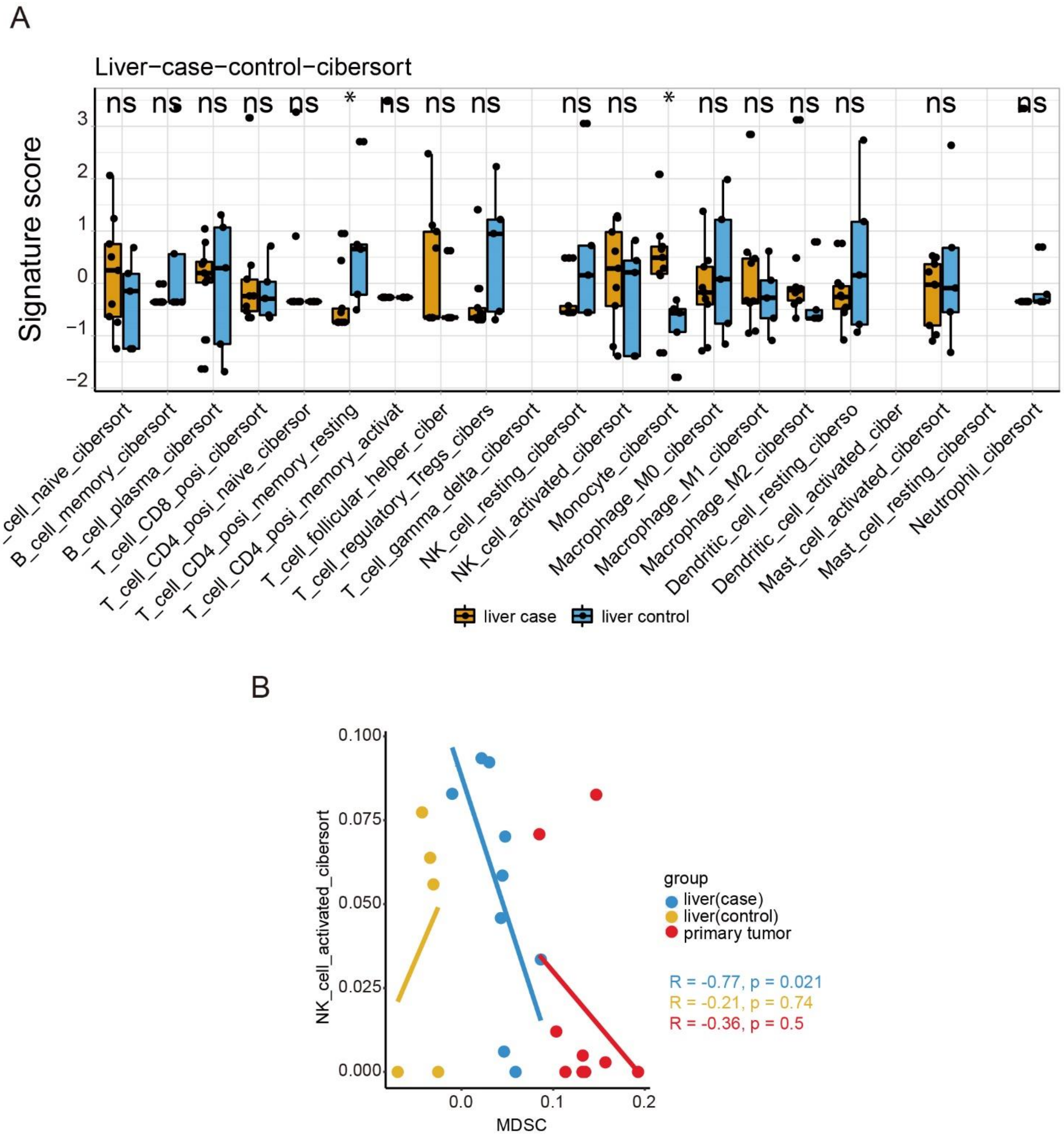

Supplementary Figure 2. Immune cell fraction of pre-metastatic liver and control liver. A. The immune cell infiltration fraction of the pre-metastatic liver and control liver. B. Scatter plots depicting the correlation between MDSC and activated NK cell signature of liver (control), liver (case), and primary tumor (case) respectively.

2.3 Supplementary Figure 3

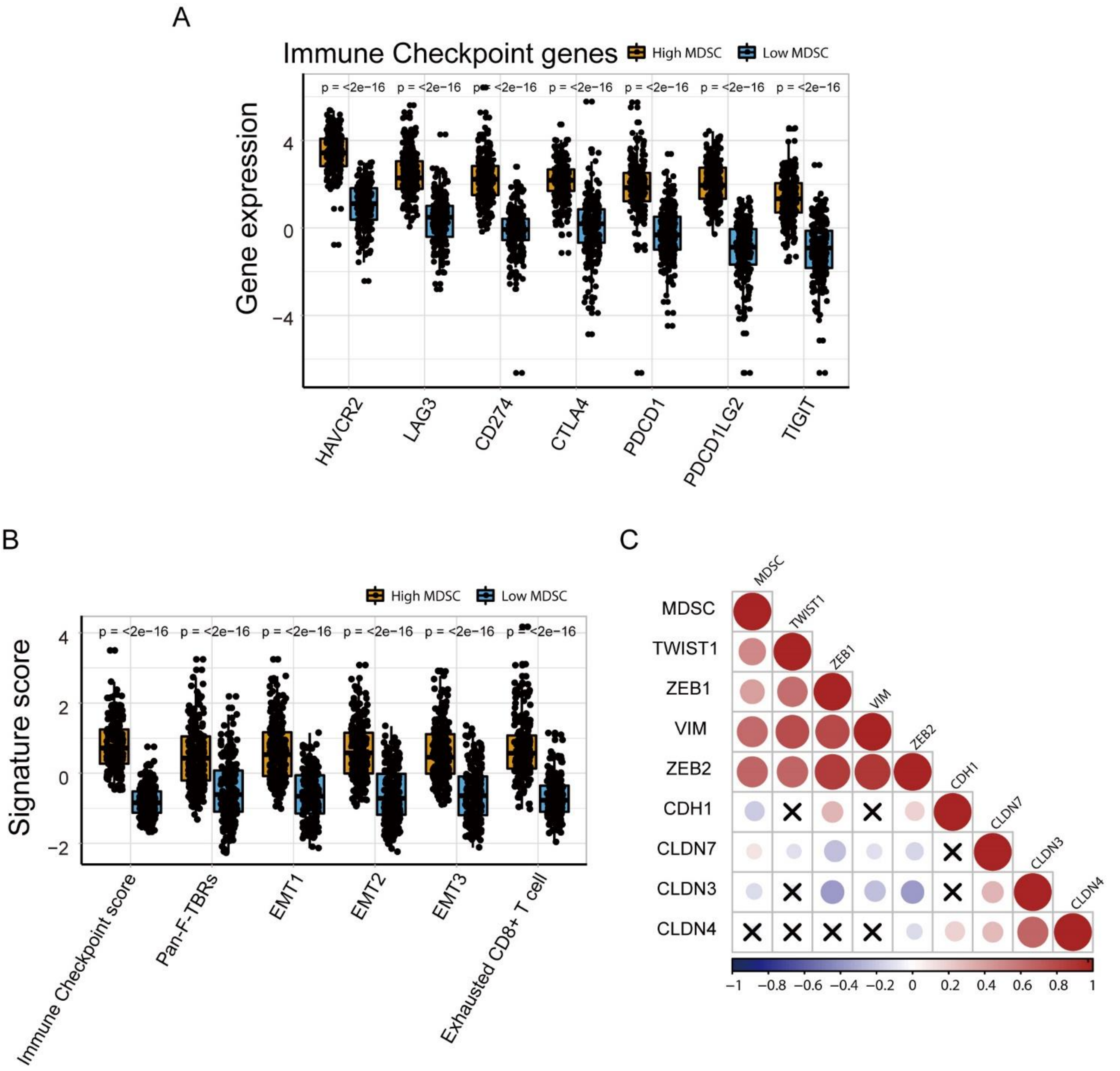

Supplementary Figure 3. MDSCs relevant immune characteristics in the COAD-READ cohort. A. Immune checkpoint genes expressed diffenrently in high MDSC and low MDSC groups. B. Immune exclusive signatures (Immune Checkpoint Score, Pan-F-TBRs, EMT1, EMT2, EMT3, and Exhausted CD8+ T cell) in high MDSC and low MDSC groups. C. Correlation matrix of MDSC and genes linked to EMT.

2.4 Supplementary Figure 4

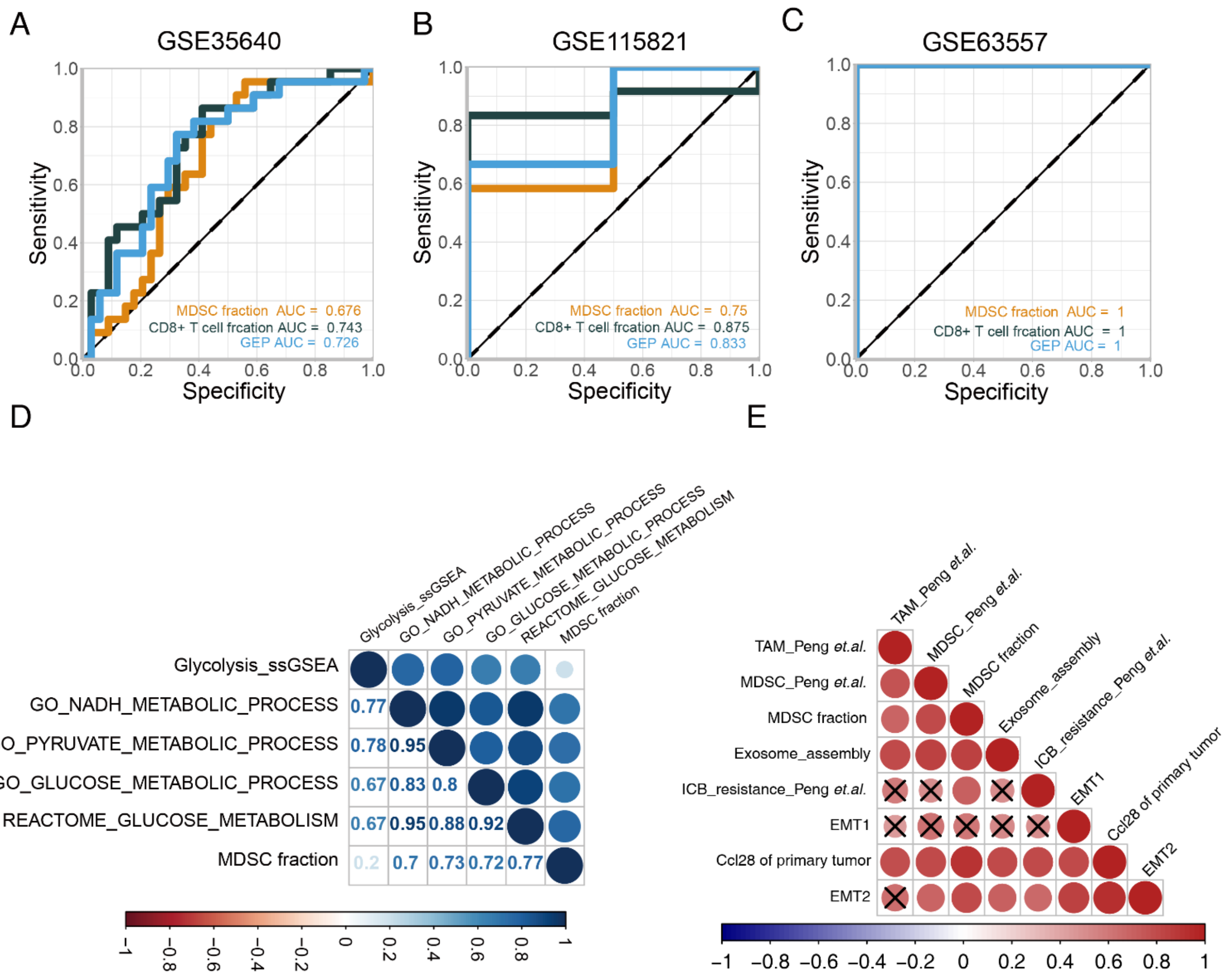

Supplementary Figure 4. Predictive value of MDSC fraction towards immune checkpoint inhibitor therapy. A. Receiver operating characteristic(ROC) curves for MDSC fraction, CD8+ T cell fraction, and GEP in the GSE35640 cohort. B. ROC curves for MDSC fraction, CD8+ T cell fraction, and GEP in the GSE115821 cohort. C. ROC curves for MDSC fraction, CD8+ T cell fraction, and GEP in the GSE63557 cohort. D. Correlation matrix of the MDSC fraction from the pre-metastatic liver and the glycolysis signature and its related signatures of the primary tumor. E. Correlation matrix of the Ccl28 of primary tumor and the immune suppressive signature score of pre-metastatic liver.
